# Analysis of Circulating Haemocytes from *Biomphalaria glabrata* following *Angiostrongylus vasorum* Infection Using Flow Cytometry

**DOI:** 10.1155/2012/314723

**Published:** 2012-03-25

**Authors:** Thales A. Barçante, Joziana M. P. Barçante, Ricardo T. Fujiwara, Walter S. Lima

**Affiliations:** ^1^Departamento de Medicina Veterinária, Pontifícia Universidade Católica de Minas Gerais (PUC Minas), 30535-901 Belo Horizonte, MG, Brazil; ^2^Departamento de Medicina Veterinária, Preventiva, Universidade Federal de Lavras, 37200-000 Lavras, MG, Brazil; ^3^Departamento de Parasitologia, Universidade Federal de Minas Gerais, 31270-901 Belo Horizonte, MG, Brazil

## Abstract

*Angiostrongylus vasorum* is an emerging parasite of dogs and related to carnivores that have an indirect life cycle, with a wide range of terrestrial and aquatic gastropods as the obligatory intermediate host. Unfortunately, the relationship between *A. vasorum* and their snail hosts remains poorly understood. Circulating haemocytes are the main line of cellular defence involved in the destruction of helminths in snails. Aiming to further characterize the haemocyte subsets in *Biomphalaria* snails, we have performed a flow cytometric analysis of whole haemolymph cellular components using a multiparametric dual colour labelling procedure. Our findings demonstrated that *B. glabrata* infected with *A. vasorum* have two major circulating haemocyte subsets, referred to as small and large haemocytes. Differences in the cell proportion occurred over time. The development of better invertebrate infection control strategies would certainly result in the better control of human diseases caused by other species of the genus *Angiostrongylus*. Such knowledge will assist in the establishment of novel control strategies aimed at parasites that use molluscs as intermediate hosts and clarify new aspects of the parasite-host relationship regarding cell recognition and activation mechanisms, which are also found in the innate response of vertebrates.

## 1. Introduction

*Angiostrongylus vasorum* is a nematode belonging to the superfamily Metastrongyloidea that parasitizes domestic dogs (*Canis familiaris*) and wild carnivores. *Angiostrongylus vasorum *is distributed throughout Europe, the Americas, and Africa [1]. Isolated endemic foci are generally observed, although reports of infections outside of these focal areas have increasingly been documented [2]. A determinant factor in the pathology of canine angiostrongylosis seems to be related to the location of the parasite in the definitive host. The presence of the parasite inside the arteries and arterial branches of the host promotes a mechanical and metabolic action on the vessel walls, which may alter homeostasis [3, 4], resulting in pneumonia, loss of racing performance, coughing, and anaemia [5]. Severely infected dogs may develop heart failure and pulmonary fibrosis, followed by weight loss, haemorrhagic diatheses, and death [6, 7].

*Angiostrongylus vasorum *has an indirect life cycle, with a wide range of terrestrial and aquatic gastropods as the obligatory intermediate host [8–13]. Some terrestrial molluscs are natural hosts of *A. vasorum*. In the laboratory, this nematode can be maintained in some species of planorbids. *Biomphalaria glabrata* is a fresh water planorbid of considerable importance as experimental intermediate host of *Angiostrongylus *sp. Species of *Biomphalaria* are capable of maintaining their integrity mainly through the activity of their internal defence system, which is made up of both cellular and soluble components. Circulating haemocytes are the main line of cellular defence involved in the destruction of helminths in snails that serve as the intermediate host [14–16]. These cells can bind to infectious agents by phagocytosing the syncytial tegument and/or releasing cytotoxic compounds [17].

Circulating haemocytes in *B. glabrata* are composed of at least two cell populations: hyalinocytes and granulocytes [16, 18–21]. These cell populations differ substantially in morphological, biochemical, and functional aspects [22, 23]. The successful elimination of potential infective agents requires granulocytes to engulf particles and further eliminate living pathogens through enzymatic or oxidative degradation. In contrast, hyalinocytes are thought to be responsible primarily for wound repair [24, 25], requiring aggregation at an injury site.

During infection by helminths, the internal defence system of species of* Biomphalaria* is capable of recognising parasite molecules through a system similar to (soluble or membrane-binding) pattern-recognition receptors and activating phagocytes, which leads to parasite encapsulation and destruction [26]. Unfortunately, the relationship between *A. vasorum *and their snail hosts remains poorly understood. The development of better invertebrate infection control strategies would certainly result in the better control human diseases caused by other species of the genus *Angiostrongylus.*

The aim of the present study was to describe the profile of circulating haemocytes in *B. glabrata* following *A. vasorum *infection using flow cytometry analysis.

## 2. Materials and Methods

### 2.1. Parasite Source

The strain of *A. vasorum* used in the experiments was isolated from the faeces of a domestic dog. The nematode was maintained by serial infections of domestic dogs and aquatic snails (*B. glabrata*) at the Laboratory of Veterinary Helminthology, Department of Parasitology, *Universidade Federal de Minas Gerais* (UFMG, Brazil).

The experimental protocols were carried out in compliance with the Ethical Principles in Animal Experimentation adopted by the UFMG Ethics Committee in Animal Experimentation and were approved under process number 060/03.

### 2.2. Parasitic Infection

First-stage larvae of *A. vasorum *(L1) were isolated from the faeces of an experimentally infected dog using a Baermann apparatus, followed by filtration using filter paper, as described by Barçante et al. [27]. The number of viable L1 larvae was estimated under a stereomicroscope (magnification: 40x). *Biomphalaria glabrata *snails were individually exposed to 1000 L1 larvae of *A. vasorum *for 24 h in 2 mL of dechlorinated water [28]. Groups of 10 individuals were kept in containers throughout the trial, maintained at room temperature (25 to 27°C), and fed on lettuce.

### 2.3. Haemolymph Collection

The whole haemolymph was collected from the snails at different times during *A. vasorum *infection: 30 min, 1, 2, 3, 4, 5, 6, 12, 24, 48, and 72 h after infection as well as 10, 20, 30, and 60 days after infection. The haemolymph was collected using the method described by Martins-Souza et al. [16]. Each snail shell was cleaned with 70% alcohol, dried with absorbent tissue paper, and the haemolymph was collected by a cardiac puncture using a 21-gauge needle. To avoid cell agglutination, the whole haemolymph was collected and diluted [1 : 1 (v/v)] in Chernin's balanced salt solution (CBSS) (47.7 mM of NaCl, 2.0 mM of KCl, 0.49 mM of anhydrous Na_2_HPO_4_, 1.8 mM of MgSO_4_ · 7H_2_O, 3.6 mM of CaCl_2_ · 2H_2_O, 0.59 mM of NaHCO_3_, 5.5 mM of glucose and 3 mM of trehalose) containing citrate/EDTA buffer (50 mM of sodium citrate,10 mM of EDTA, and 25 mM of sucrose) at pH 7.2.

### 2.4. Haemocyte Count

The haemolymph from three snails of each time group was divided in triplicate (1 mL) in microtubes and tested. After debris sedimentation for two min, the whole haemolymph was transferred to another microtube (1 mL). Total haemocyte counts were performed using 10 *μ*L of whole haemolymph diluted (1/10) in CBSS buffer containing 0.5% neutral red solution [neutral red retention assay (NRRA)]. Haemocytes that stained red were considered granulocytes and those that did not were considered hyalinocytes.

### 2.5. Flow Cytometry Analysis

Flow cytometry analysis was performed as described by Martins-Souza et al. [16]. After individual collection, the haemolymphs from three snails from the same experimental group were pooled and three separate pools were prepared and tested for each experimental group.

Haemolymph incubation with propidium iodide (PI) and acridine orange (Ao) solution allowed the separation of the viable circulating haemocytes (Ao positive/Eb negative cells) from the dead cells (Ao negative/Eb positive cells) and small fragments (Ao and Eb negative events). A total of 150 *μ*L of the pooled whole haemolymph was mixed with an equal volume of propidium iodide (25 mg of PI/mL 95% alcohol) and acridine orange (7.5 mg of Ao/mL 95% alcohol) diluted (1 : 1000) in CBSS citrate/EDTA.

Haemocyte suspension was incubated for one h on ice in the absence of light. The suspension was then analysed using a FACScan flow cytometer (BD—Becton Dickinson, USA). Flow cytometry analysis of the cellular components of the whole haemolymph was performed using instrument settings to capture the fluorescence signals from propidium iodide (FL2) and acridine orange (FL3), using log amplification scales. A total of 15,000 events were analysed for each pooled haemolymph sample.

Data analysis was performed using the Cell Quest program (Becton Dickinson, USA). FL2 versus FL3 dot plot distribution graphs were constructed to differentiate live cells from dead cells and debris. Live haemocytes were selected based on size versus internal complexity (laser forward scatter (FSC) and side scatter (SSC), resp.). Two major haemocyte subpopulations were selected based on FSC. Percentages of haemocyte subsets obtained from the flow cytometry analysis were further converted into absolute counts taking into account the total viable haemocyte counts (NRRA) performed in a Neubauer chamber obtained with the same haemolymph sample. 

### 2.6. Statistical Analysis

Data referring to the numbers of circulating haemocytes within each cell subset are reported as mean and standard deviation and analysed using one-way analysis of variance (ANOVA) followed by the Bonferroni post test.

## 3. Results

**Figure 1:**
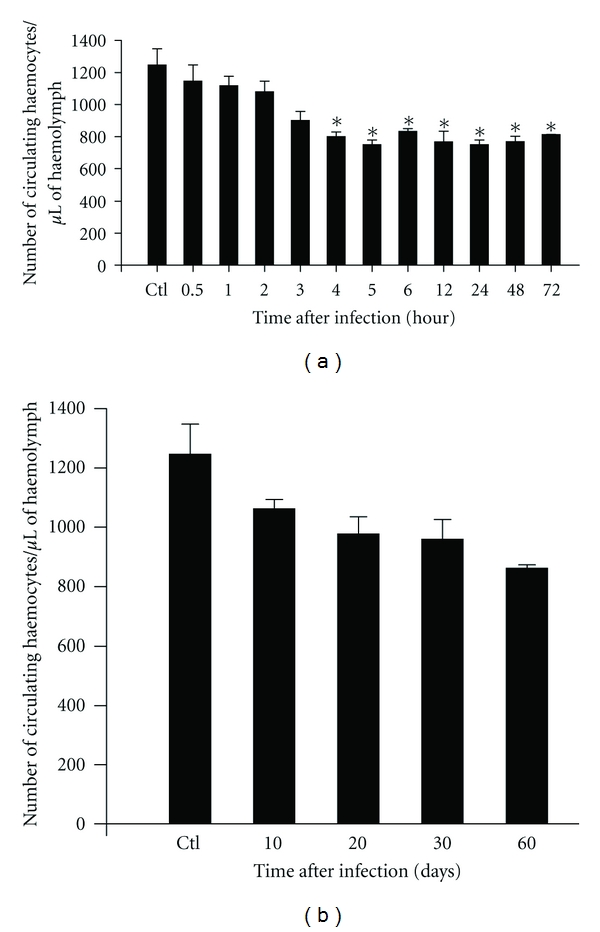
Mean number of circulating haemocytes/*μ*L of haemolymph in three snails from each experimental group. Ctl—*Biomphalaria glabrata *free from infection with *A. vasorum *and *B. glabrata *exposed to 1,000 first-stage larvae of *A. vasorum *at different time of infection. Data are presented as mean number ± standard deviation of circulating haemocyte subpopulations. (*) Represents significant differences (*P* < 0.05) in the number of haemocytes of each point of infection to control group.

During the course of infection, a significant reduction occurred in the number of circulating haemocytes from four to 72 hours after exposure to *A. vasorum* L1 larvae (*P* < 0.05) (Figure 1(a)). This reduction was gradual through five hours after infection. From six to 72 hours after infection, the number of circulating haemocytes in the snails remained without much alteration, although at a significantly lower number than that in the control group (*P* < 0.05). From 10 to 60 days after infection, normal patterns of total haemocytes were reestablished in the haemolymphs, with no significant differences between groups (*P* > 0.05) (Figure 1(b)).

**Figure 2:**
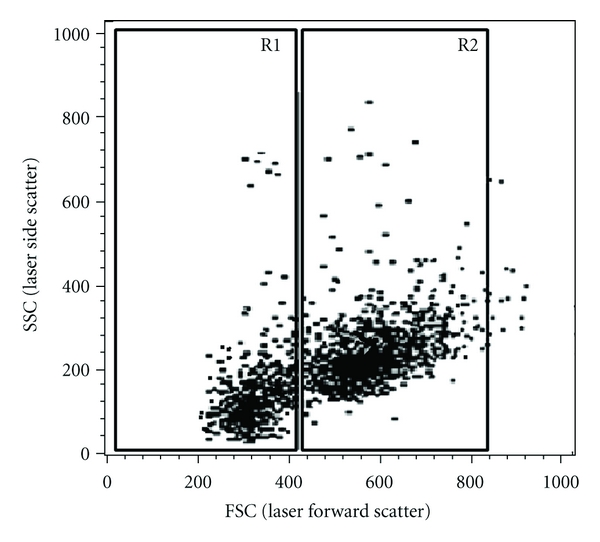
Profile of circulating haemocyte population in *Biomphalaria glabrata *snails. Two haemocyte subpopulations (R1 = small—FSC between 200–400, R2 = large—FSC between 440–840 can be identified by flow cytometric dot plot distributions based on their laser forward scatter (FSC) versus laser side scatter properties (SSC).

Haemolymph incubation with propidium iodide and acridine orange solution allowed the separation of viable circulating haemocytes from dead haemocytes (Figure 2). Viable circulating haemocytes from *Biomphalaria* snails were separated into two major cell subpopulations based mainly on size (small (FSC channels between 200 and 440) and large (FSC channels between 440 and 840)) and granularity. The haemocyte subpopulations were denominated small (R1) and large (R2). 

**Figure 3:**
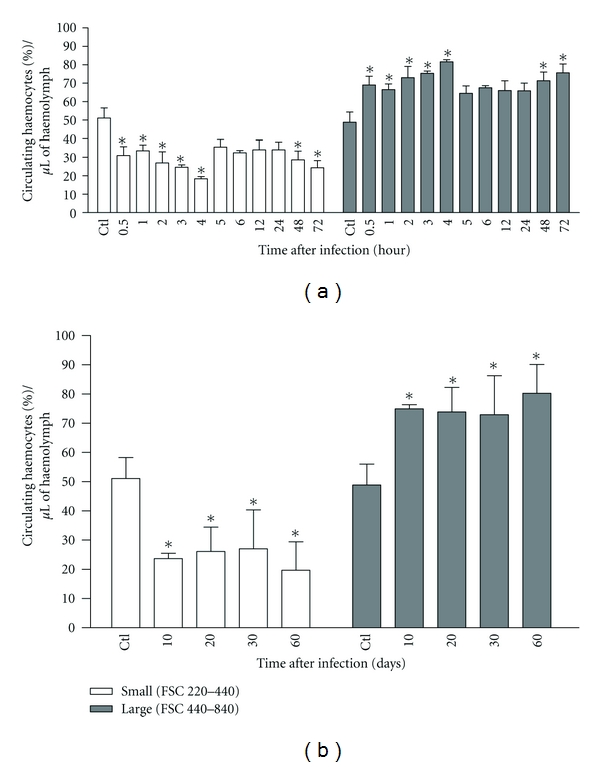
Percentual number of small (R1) and large (R2) haemocyte subpopulations in 1 *μ*m of haemolymph of *Biomphalaria glabrata *free from infection with *Angiostrongylus vasorum *(Ctl) and in 1 *μ*m of haemolymph of *B. glabrata *exposed to 1,000 first-stage larvae of *A. vasorum* at different times of infection. Data are presented as mean number ± standard deviation of circulating haemocyte subpopulations. (*) Represents significant differences (*P* < 0.05) in the number of haemocytes at each point of infection for the control group.

In the control, 41% of the haemocytes were small and 59% were large (Figure 3). Thirty minutes after infection, there was a significant reduction in the percentage of small circulating haemocytes. This phenotype profile remained constant until four hours after infection (Figure 3(a)). In the subsequent hours, the number of small cells demonstrated a tendency toward a reduction until reaching significantly lower values than those of the control at 48 and 72 hours (*P* < 0.05). From 10 to 60 days after infection, the number of small haemocytes recovered in the infected snails remained below that found in the control group (*P* < 0.05) (Figure 3(b)).

## 4. Discussion

Although invertebrates account for the vast majority of living beings, publications addressing defence mechanisms against pathogens have been restricted mainly to interactions with pathogens of vertebrate animals [15]. Since the description of the participation of molluscs as intermediate hosts in the evolutional cycle of *A. vasorum *by Guilhon [29], very little has been revealed regarding the infection biology and parasite-host interaction, especially in relation to the intermediate host. Studies demonstrate differences in the susceptibility of some species of molluscs to infection by *A. vasorum* [13, 30].

The capacity of molluscs to respond strongly to stimuli depends on haemocyte viability and functional capacity. Studies carried out on *B. tenagophila* demonstrate that the temporary reduction in the number of circulating granulocytes results in increased susceptibility to infection by *Schistosoma mansoni* [23].

In the present study, the number of circulating haemocytes in the haemolymph of *B. glabrata *reduced significantly soon after infection by *A. vasorum* through to 72 hours after infection. As the circulatory system of molluscs is generally open, haemocytes can move freely in and out of the tissues [31]. In histological cuts of *B. glabrata* infected by *A. vasorum*, Barçante [32] found that a perilarval cellular infiltrate occurs in the tissues five hours after infection. Similarly, Bezerra et al. [33] report a reduction in the population of circulating haemocytes in *B. glabrata *in the first five hours after infection by *S. mansoni. *These observations indicate that, in infections of *B. glabrata *by *A. vasorum*, with the onset of a perilarval tissue reaction and the formation of a typical granuloma, circulating cells migrate to the tissue, leading to a reduction in the amount of these cells in the haemolymph.

Over the course of the infection, the chemotaxis of cells to the site of larval infection ceases, with a consequent reestablishment of cell levels, as seen beginning 10 days after infection [32]. According to Barçante [32], there is a reduction in perilarval cellularity in the long term, which explains the stabilisation in the cell composition of the haemolymph beginning 10 days after infection. Similarly, Pereira et al. [23] report an increase in haemocyte activation with no corresponding increase in the number of circulating haemocytes in *B. tenagophila *infected by *A. vasorum* due to the migration of circulating cells to the infection site during the encapsulation process.

The haemocytes of other molluscs and invertebrates are generally rather easy to define morphologically and to distribute among categories of known functions. In contrast, the haemocytes in *B. glabrata *do not offer simple criteria for recognition, such as specific secretory granules [34]. Thus, flow cytometry analysis proved to be a useful tool for the analysis of circulating haemocytes. Specifically in *B. glabrata*, two subpopulations of cells were characterized in noninfected snails: small round cells ranging from 5 to 6 *μ*m in diameter with little granularity and large cells ranging from 6.5 to 8 *μ*m in diameter with a greater degree of granularity [22].

The present study found two subpopulations with similar characteristics to those described previously as well as changes in the cell profile during infection by *A. vasorum*. Differences in the cell proportion occurred over time. A number of studies demonstrate that hyalinocytes are precursors of granulocytes and that, in cytometric counts, small cells are hyalinocytes and small granulocytes, whereas large cells are granulocytes. Thus, with the onset of infection by *A. vasorum, *the proportion of small cells diminishes due to the conversion of hyalinocytes into granulocytes, with the subsequent migration of these granulocytes to the tissues after five hours (as seen in histological cuts), thereby explaining the increase in the proportion of small cells in the haemolymph at this point [32]. This finding corroborates the results described by Bezerra et al. [33], who explain the reduction in the number of granulocytes in the haemolymph by the fact that granulocytes are effecter cells that migrate to the infection site, as demonstrated by histological analysis. In the later stages of infection, the percentages of small cells are lower due to the fact that the mollusc is infected and the effecter cells are acting, even at a lesser intensity, as seen in histological cuts [32].

## 5. Conclusion

The study of the parasite-host relationship by evaluating of the infection of *B. glabrata *by *A. vasorum *has much to contribute toward knowledge on the mechanisms used by the internal defence system of these invertebrates. Such knowledge will assist in the establishment of novel control strategies aimed at parasites that use molluscs as intermediate hosts and clarify new aspects of the parasite-host relationship regarding cell recognition and activation mechanisms, which are also found in the innate response of vertebrates. Moreover, *A. vasorum *is a nematode of increasing veterinary importance for which the possibility of being a causal agent of zoonoses has not yet been discarded, making studies on the interaction between molluscs and parasites important to the understanding of the biology and epidemiology of angiostrongylosis.
